# Draft Genome Sequences of Seven Extended-Spectrum β-Lactamase-Producing Escherichia coli Strains Isolated from New Zealand Waterways

**DOI:** 10.1128/MRA.01445-20

**Published:** 2021-03-18

**Authors:** Sara A. Burgess, Margaux Francois, Anne C. Midwinter, Patrick J. Biggs

**Affiliations:** a *^m^*Epilab, School of Veterinary Science, Massey University, Palmerston North, New Zealand; b School of Fundamental Sciences, Massey University, Palmerston North, New Zealand; University of Maryland School of Medicine

## Abstract

Draft genomes of seven extended-spectrum β-lactamase (ESBL)-producing Escherichia coli strains recovered from New Zealand waterways are described. The mean genome size was 5.1 Mb, with 4,724 coding sequences. All genomes contained the ESBL gene *bla*_CTX-M_, and one carried a plasmid-mediated AmpC gene, *bla*_CMY-2_. A multidrug-resistant genotype was detected in three isolates.

## ANNOUNCEMENT

Extended-spectrum β-lactamase (ESBL)-producing Escherichia coli strains are commonly associated with multidrug-resistant urinary tract infections (1, 2). An important pathway for the spread of antimicrobial-resistant bacteria is person-to-person transmission (3), but other transmission pathways, including contaminated waterways, may also be relevant (4–6).

Mixed cellulose ester filters (0.45 μm; Millipore, Germany) from 100-ml samples of water from two storm water drains (Meola Reef Park and Northboro Reserve, Auckland, New Zealand) and one stream (Momutu Stream, Auckland, New Zealand) were enriched in 10 ml of buffered peptone water (BD Difco, Becton, Dickinson, Heidelberg, Germany) and subcultured on MacConkey agar (BD Difco; supplied by Fort Richard Laboratories, Auckland, New Zealand) or ChromESBL (CHROMagar, Paris, France; supplied by Fort Richard Laboratories), and single colonies were purified on Columbia horse blood agar (Fort Richard Laboratories). DNA extractions were performed using the QIAamp DNA minikit (Qiagen, Hilden, Germany); each isolate was cultured on Columbia horse blood agar overnight at 35°C, and approximately three colonies were resuspended in 180 μl ATL buffer according to the manufacturer’s instructions. Libraries were prepared using the Nextera XT DNA library preparation kit (Illumina, San Diego, CA) and submitted to Otago Genomics Ltd. (University of Otago, Dunedin, New Zealand) for sequencing using the Illumina HiSeq platform with 2 × 125-bp paired-end reads. The read quality was assessed using FastQC (v.0.11.9). The reads were processed using the default settings in the Nullarbor pipeline (v. 2.0.20181010) (https://github.com/tseemann/nullarbor), in which trimming was carried out using Trimmomatic (v.0.39), assembly was carried out using SKESA (v.2.3.0), and annotation was carried out using Prokka (v.1.13.3) (7–9). Sequencing, assembly, and genome statistics are presented in Table 1.

**TABLE 1:** Strain information and genome statistics

Attribute	Information for strain:						
	SB0062c	SB0062f	SB0062i	SB0098e	SB0114e	SB0114f	SB0114h
Sampling date	24 September 2016	24 September 2016	24 September 2016	27 November 2016	14 January 2016	14 January 2016	14 January 2016
Sampling location	Northboro Reserve	Northboro Reserve	Northboro Reserve	Meola Reef Park	Momutu Stream	Momutu Stream	Momutu Stream
Genome length (bp)	5,101,109	5,103,614	5,208,896	4,900,256	5,125,337	5,126,052	5,155,736
No. of reads	3,394,516	4,106,836	4,022,156	3,499,930	3,819,686	4,443,052	3,940,708
No. of contigs	62	66	61	74	101	94	113
*N*_50_ (bp)	346,480	346,480	328,342	160,995	150,156	150,169	158,652
Depth (×)	82	99	97	84	92	107	95
No. of CDSs	4,708	4,699	4,827	4,562	4,723	4,719	4,831
GC content (%)	50.5	50.6	50.7	50.4	50.9	50.7	50.6
GenBank accession no.	JADDIY000000000	JADDIZ000000000	JADDJA000000000	JADDJB000000000	JADDJC000000000	JADDJD000000000	JADDJE000000000
SRA accession no.	SRR13257574	SRR13257573	SRR13257572	SRR13257571	SRR13257570	SRR13257569	SRR13257568
BioSample no.	SAMN16480291	SAMN16480292	SAMN16480293	SAMN16480294	SAMN16480295	SAMN16480296	SAMN16480297
Sequence type	4553	4553	4553	156	38	38	648
ESBL/AmpC type(s)	CTX-M-15	CTX-M-15	CTX-M-15, CMY2	CTX-M-14	CTX-M-14	CTX-M-14	CTX-M-15
Other AMR genes	ND^*a*^	ND	ND	*tetB*	*aph(3″)-Ib*, *aph(6)-Id*, *sul2*	*aph(3″)-Ib*, *aph(6)-Id*, *sul2*	*aph(3″)-Ib*, *aph(6)-Id*, *sul2*

a ND, not detected.

The mean genome size was 5.1 Mb, with an average GC content of 50.6% and 4,724 coding sequences (CDSs). Antimicrobial resistance (AMR) genes were identified using ResFinder (v.3.1) (10). The seven isolates all contained the ESBL gene *bla*_CTX-M_, and one isolate also carried a plasmid-mediated AmpC gene, *bla*_CMY-2_. A multidrug-resistant genotype (AMR genes associated with three or more classes of antibiotics) was detected in the three strains isolated from the Momutu Stream. This study reinforces the need to take a holistic “One Health” approach to understanding the sources and transmission pathways for the community spread of antimicrobial-resistant bacteria.

### Data availability.

The draft genome assemblies have been deposited in GenBank, and their accession numbers are detailed in Table 1.
